# Are We Ready for Pseudotumors in Total Ankle Arthroplasty? A Case Report

**DOI:** 10.3390/jcm14020649

**Published:** 2025-01-20

**Authors:** Federico Sgubbi, Antonio Mazzotti, Alberto Arceri, Simone Ottavio Zielli, Elena Artioli, Laura Langone, Marco Gambarotti, Cesare Faldini

**Affiliations:** 1 Department of Biomedical and Neuromotor Sciences (DIBINEM), Alma Mater Studiorum University of Bologna, 40123 Bologna, Italy; federico.sgubbi@ior.it (F.S.); antonio.mazzotti@ior.it (A.M.); simoneottavio.zielli@ior.it (S.O.Z.); elena.artioli@ior.it (E.A.); laura.langone@ior.it (L.L.); cesare.faldini@ior.it (C.F.); 2 1st Orthopaedics and Traumatologic Clinic, IRCCS Istituto Ortopedico Rizzoli, 40136 Bologna, Italy; 3 Department of Pathology, IRCCS Istituto Ortopedico Rizzoli, 40136 Bologna, Italy; marco.gambarotti@ior.it

**Keywords:** total ankle arthroplasty, pseudotumor, hypersensitivity, debris wear

## Abstract

**Background:** Pseudotumors are defined as exuberant non-neoplastic inflammatory masses. This condition can be associated with hip and knee arthroplasty but has not been reported in Total Ankle Arthroplasty (TAA). This paper reports a pseudotumor that formed following TAA, highlighting its clinical presentation, management, and histopathology. **Methods:** A 55-year-old male with end-stage post-traumatic ankle osteoarthritis underwent TAA using a mobile-bearing prosthesis. The procedure was reported to be successful, with no immediate complications. **Results:** Three years postoperatively, following a period of symptom resolution, the patient presented with progressively worsening ankle pain, swelling, and limited weight-bearing ability. Imaging revealed indirect signs of a periarticular mass and loosening components. Revision surgery involved prosthesis explantation and mass excision for histological and microbiological analysis, followed by concomitant tibio-talo-calcaneal fusion with a retrograde nail. The histopathology identified a pseudotumor characterized by chronic inflammation, fibrous tissue, and necrotic debris, with no evidence of infection. The postoperative recovery was uneventful, with pain resolution and successful fusion confirmed at a one-year follow-up. **Conclusions:** In patients experiencing unexplained pain or symptoms following TAA, the possibility of a pseudotumor, although rare, should be considered. Prompt and comprehensive clinical and radiographic evaluation is crucial to raise suspicion and prevent this condition from being overlooked.

## 1. Introduction

Total Ankle Arthroplasty (TAA) is a well-established treatment for patients with end-stage ankle osteoarthritis that has been demonstrated to offer satisfactory results in terms of clinical and functional outcomes [1]. Despite technological developments and improvements in prosthetic designs and materials [2,3,4,5], the literature continues to report high numbers of revisions and complications associated with TAA. A recent systematic review and meta-analysis of 127 studies identified 30 distinct potential complications following TAA [6]. However, pseudotumors have not been documented. In contrast to TAA, pseudotumor formation is a well-documented complication in hip and knee arthroplasty. This condition, defined as a non-neoplastic inflammatory mass within the periprosthetic tissue, is frequently associated with pain, periprosthetic bone loss, and loosening of prosthetic components [7].

The precise incidence of this pathology remains uncertain and varies significantly across anatomical sites [8]. In hip and knee arthroplasty, the frequency and histological presentation of pseudotumors appear to correlate with high wear and metal hypersensitivity (cell-mediated, delayed, or type IV hypersensitivity) [9,10,11].

A randomized control trial observed an overall pseudotumor incidence of 54% in Metal-on-Metal (MoM) Total Hip Arthroplasty (THA) compared with 21.8% in Metal-on-PolyEthylene (MoPE) THA at a medium-term follow-up of 50 months [12]. In the context of Total Knee Arthroplasty (TKA) [9,13,14,15], a recent review reported a pseudotumor incidence of 1.6% [16].

To date, as far as we are aware, no instance of a pseudotumoral reaction similar to those observed in hip and knee arthroplasty has been reported in TAA. Previous studies included cases of prosthetic-released debris, which led to modest inflammatory reactions with cyst formation and implant failure [17,18]. Although less common, hypersensitivity reactions to metal implants, which generally manifest with local and systemic symptoms [19], have also been documented in the literature [20,21]. Some studies reported post-TAA hypersensitivity reactions involving different metal alloys, particularly those containing nickel, cobalt, and chromium, which led to osteolysis or loosening of the components and implant failure without pseudotumor mass formation [22].

The aim of this study is to present the case of a 55-year-old male patient who developed an exuberant reaction in the form of a non-neoplastic inflammatory mass around the periprosthetic tissue, referred to as a pseudotumor, three years after TAA.

## 2. Case Report

A 55-year-old male with end-stage post-traumatic osteoarthritis of the left ankle, resulting from a severe tibial pilon fracture with medial dislocation, a diaphyseal fibular fracture, and multiple foot fractures, was treated with TAA using a BOX^®^ Total Ankle Replacement (MatOrtho Limited, Leatherhead, UK), a three-component mobile-bearing prosthesis [23]. Three years after the procedure, the patient came to our attention reporting new-onset ankle pain, severe weight-bearing impairment, and daily activity limitations despite ongoing medical and physical therapies. A clinical examination revealed healthy skin and scar tissue, with no evidence of local infection. However, the ankle was observed to be swollen. Pain was localized around the medial malleolus and the anterior prosthetic area.

**Figure 1:**
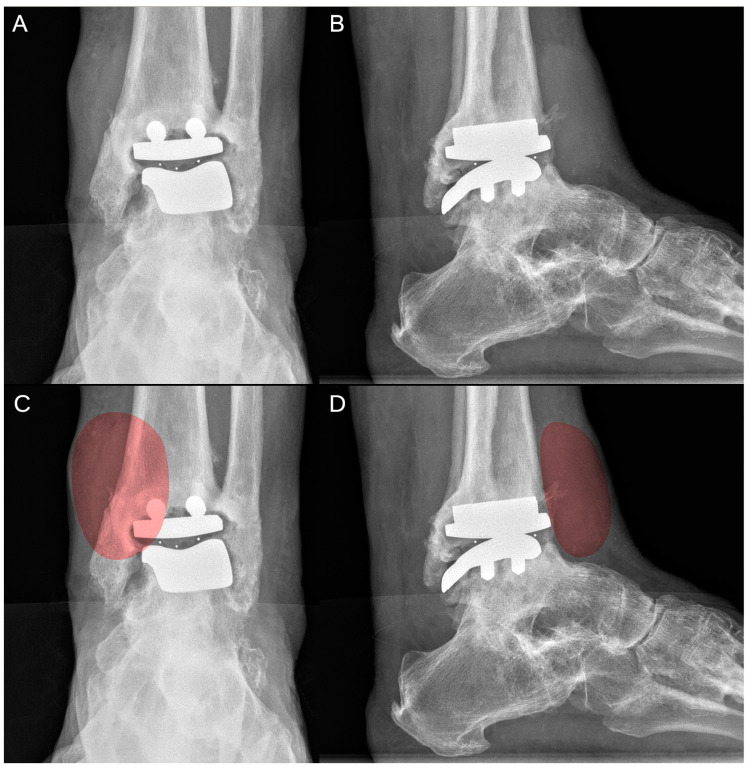
Radiographic evaluation revealed mild resorption at the prosthesis–bone interface, accompanied by signs of tibial and talar component loosening and alterations in the physiological bone contour (**A**). A periarticular mass extended medially and anteriorly from the tibial component (**B**). The mass was identified, and it is highlighted in red in both the anteroposterior (**C**) and lateral (**D**) views.

Radiographic evaluation revealed mild bone resorption at the prosthesis interface with early signs of tibial and talar loosening medially and anteriorly. Additionally, a periarticular mass was observed extending anteriorly from the tibial component, with diffuse intra- and perilesional ossifications (Figure 1).

The local clinical presentation, with a good general condition and absence of fever, did not indicate an infection. Moreover, blood tests, including the leukocyte count, Erythrocyte Sedimentation Rate (ESR), and C-Reactive Protein (CRP), were performed, and all results were within the normal ranges. A differential diagnosis of septic loosening was considered but ruled out in the first instance due to the absence of clinical signs of local or systemic inflammation and negative inflammatory markers, including the ESR and CRP. Nevertheless, a preoperative infectious disease consultation was performed. This consultation excluded a septic etiology, supported the planned surgical approach, and recommended obtaining biopsy samples for histological and microbiological examination. No indication was provided regarding the necessity of postoperative antibiotic therapy.

Neoplastic degeneration was not considered due to the absence of a history of tumors in the patient’s medical history, the radiographic presentation showing no signs of tumor degeneration, and the close spatial and temporal association between the mass and the prosthesis.

The condition was initially interpreted as aseptic loosening. A computed tomography (CT) scan was proposed; however, the patient declined further diagnostic investigations. Considering that there was no evidence of severe bone loss, the CT scan was not performed.

Given the progressive functional deterioration and the absence of signs of infection, a decision was made to explant the prosthetic components and perform a concomitant tibio-talo-calcaneal fusion using a retrograde intramedullary nail made of type II anodized titanium alloy (Ti6Al4V), a hypoallergenic metal. This decision was made in agreement with the patient, who refused a TAA revision procedure.

**Figure 2:**
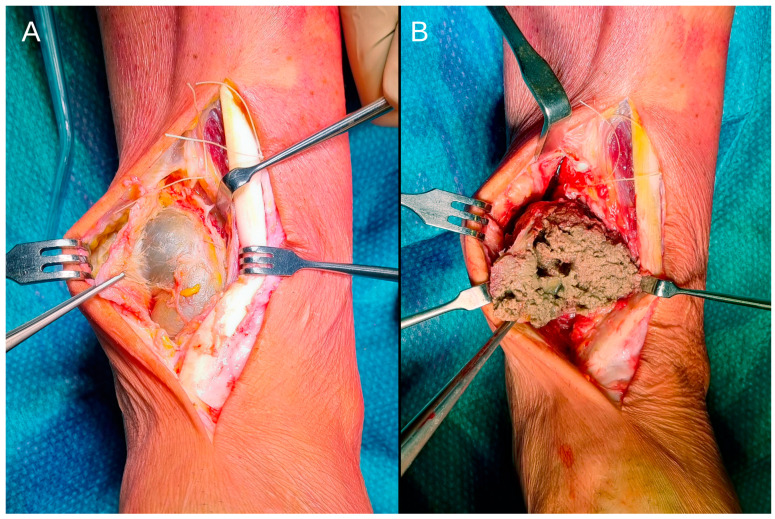
A tenso-elastic mass (**A**) was observed, which contained a brownish-grey granular substance (**B**).

The surgical approach was made along the previous incision. Upon reaching the deep muscle plane, a tenso-elastic mass was identified. It was not adherent to the superficial or deep tissues and was connected to the tibial prosthetic component. The mass contained a brownish-grey granular substance (Figure 2).

During the surgery, no signs of infection were observed in the tissues, and three intraoperative histological examinations of frozen sections were negative for infection. The tibial and talar components were explanted and sent to the laboratory for culture after sonication, along with five additional samples for culture and two for histological examination. The lesion was excised and sent for histological and microbiological analysis. A tibio-talo-calcaneal fusion was performed using a retrograde nail.

The postoperative course was uncomplicated, with no evidence of infection at the surgical site. Ten days after the surgical procedure, the patient was permitted to ambulate with a weight-bearing leg cast, limiting the load to 30 kg. Following a one-month period, the patient began to gradually increase weight bearing and demonstrated functional improvement.

At serial 1-, 2-, and 6-month follow-ups, the patient reported significant pain relief. Radiological assessments conducted during each visit demonstrated no evidence of pseudotumor recurrence and confirmed successful and complete ankle fusion.

**Figure 3:**
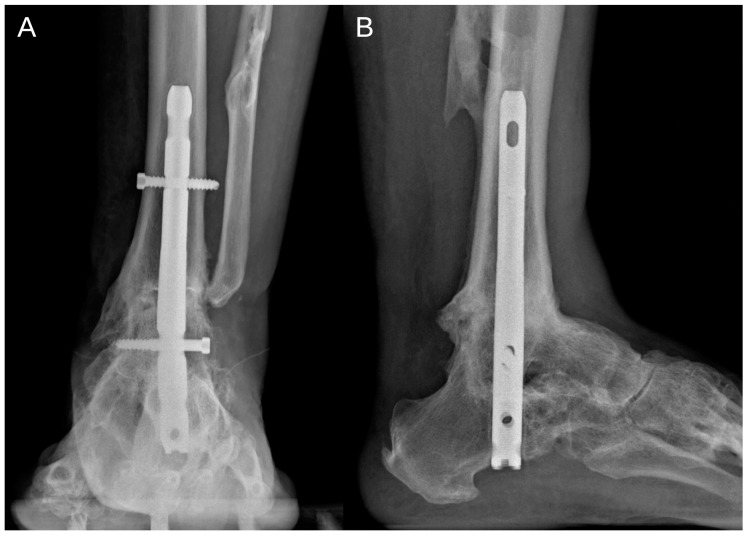
Radiographic studies demonstrated successful fusion of the tibiotalar and subtalar joints in a neutral position, as observed in the anteroposterior (**A**) and lateral (**B**) views.

At the 1-year follow-up, there was complete resolution of pain and significant clinical improvement. Radiographic studies confirmed successful fusion of the tibiotalar and subtalar joints in a neutral position (Figure 3).

**Figure 4:**
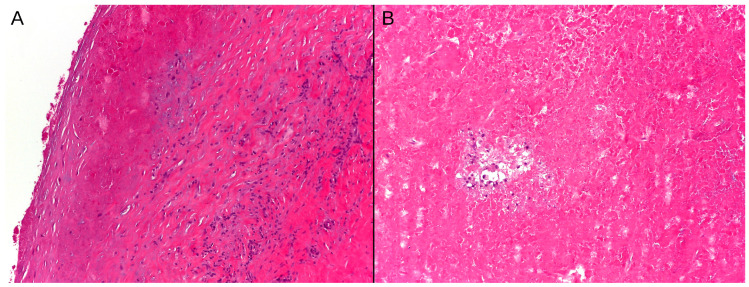
Histological slides examined under a microscope revealed fibrin and necrotic debris associated with fibrous tissue exhibiting chronic inflammatory infiltration and a fibro-histiocytic reaction (**A**). Additional findings included fibrin and necrotic debris with scattered histiocytes (**B**).

All intraoperative cultures were negative. Biopsy samples analyzed by a pathologist showed the presence of fibrin and necrotic debris associated with fibrous tissue showing chronic inflammatory infiltration and a fibro-histiocytic reaction, with scattered histiocytes (Figure 4). Both samples lacked evidence of acute inflammation, excluding the infective process.

## 3. Discussion

This case report documents a patient affected by a pseudotumor three years after TAA.

### 3.1. Diagnosis

The diagnosis of a pseudotumor was based on the clinical presentation, imaging studies, laboratory test abnormalities, and histological examination.

According to the literature, pseudotumors at other anatomical sites, such as the hip, can be asymptomatic in 4–61% of patients and can be incidental findings on imaging [12,24,25]. When symptomatic, as observed in this case, pseudotumors are usually associated with the destruction of periprosthetic bone and soft tissues [26], and they require revision surgery or amputation in the majority of patients [8].

The mass, which is typically detectable in the advanced stages as an osteolytic lesion on X-ray, can be more precisely characterized using a CT scan to evaluate its morphology, composition (cystic or solid), and bone involvement and plan surgery. MRI provides additional detail regarding its size, extent, and relationship with surrounding soft tissues [27]. As demonstrated in this case, advanced imaging can be particularly valuable when prosthetic loosening is suspected.

Laboratory blood tests may identify blood metal ion levels. Specifically, a study demonstrated that an increase in the blood cobalt level > 2.4 μg/L and a cobalt-to-chromium ratio (Co/Cr) ≥ 1.4 were predictors of pseudotumors associated with MoM hip prostheses [28]. However, in the present case, blood metal ion levels were not evaluated. They could have been contributing factors, given the presence of cobalt and chromium in the prosthetic components.

From a histological perspective, pseudotumors are characterized by an inflammatory immune response, including macrophages containing phagocytosed metal particles, as well as an adaptive immune response marked by aggregates of T lymphocytes [29,30]. Additionally, they are characterized by extensive necrosis, vascular changes, and synovial proliferation.

### 3.2. Terminology and Pathogenesis

The term “pseudotumor” may lead to confusion due to its unclear etiology and ambiguous histological nature. While some authors have suggested that a pseudotumor is a reaction to high wear [8,31], other studies have provided evidence supporting a stronger association with metal hypersensitivity [11,32]. The theory is that pseudotumor formation represents an adverse reaction associated with an immune-mediated response [33]. Whether this is immunologically mediated (a delayed hypersensitivity reaction or a type IV reaction to metal particles), a cytotoxic response to particles, or both and which factor plays the dominant role are still unclear [11].

Historically, pseudotumors were thought to be an immune response to metal wear debris, which is more common in MoM hip replacements and correlated with soft tissue destruction [8,26]. However, other conditions similar to pseudotumors have been introduced in the literature, such as metallosis, debris disease, and Aseptic Lymphocyte-Dominated Vasculitis-Associated Lesions (ALVALs), but the terminology distinction remains blurred.

Metallosis is defined as a pathological condition caused by the accumulation of metal debris in the periprosthetic tissues, particularly in MoM hip prostheses. It results from wear, corrosion, and the breakdown of implant materials, which release metal ions, in particular cobalt and chromium, into surrounding tissues, leading to local tissue necrosis and discoloration [34]. Langton et al. [35] identified higher concentrations of metal ions in patients with MoM implants, which correlated with metallosis and adverse tissue reactions.

Debris disease refers to osteolysis and inflammation caused by particulate debris from implant materials (metal, polyethylene, or ceramic). Some studies [36,37] have discussed the role of wear particles in osteolysis, highlighting how polyethylene debris triggers an inflammatory response with macrophage activation, leading to bone resorption around implants.

ALVALs represent a specific immune-mediated reaction characterized by perivascular lymphocyte infiltration in the tissue surrounding the implant. Willert et al. [38] were among the first to identify this reaction, which they associated with metal hypersensitivity. Campbell et al. [29] further refined the understanding of ALVALs, linking them to hypersensitivity rather than high wear rates alone.

In this context, the term “pseudotumor” refers to a macroscopic clinical manifestation resulting from various enteropathogenic mechanisms, such as metallosis, debris disease, or metal hypersensitivity. These mechanisms can present through diverse histopathological patterns, including ALVALs. However, other studies have distinguished metal hypersensitivity reactions with local signs but prevalent systemic allergy-like symptoms (skin reactions, dyspnea, and perioral swelling) from the clinical presentation of pseudotumors [20,21] (Table 1).

### 3.3. Pseudotumor-Related Factor

Regarding THA, pseudotumors were frequently associated with MoM hip prostheses, while those arising from MoPE hip prostheses were much rarer [39,40,41]. Cooper et al. [42] hypothesized that the formation of pseudotumors in MoPE couplings was due to corrosion at the taper in the femoral head–neck junction or edge wear, according to Harris’s study [43]. In these cases, the clinical presentation of the pseudotumors and blood test abnormalities were similar to the local adverse tissue reactions observed in patients with MoM couplings. Cases of pseudotumor have also been documented in ceramic-on-ceramic (CoC) hip prostheses [44,45], but no damage or alteration of the prosthetic elements was documented, highlighting a significant knowledge gap in the field of tribology and individual immune response mechanisms [46].

Also, for TKA, cases of metallosis were reported in different types of prostheses, which led to revision surgeries on the implants [47]. In a review of 321 non-infectious patients undergoing unilateral revision knee arthroplasty, five pseudotumors (1.6%) were identified [16]. The traditional use of polyethylene as a bearing surface in TKA prevents direct metal articulation. Thus, pseudotumor formation in TKA usually suggests atypical metal interactions between the prosthetic components [48]. Indeed, a pseudotumor was documented in association with an endoprosthetic hinge TKA, arising from metal wear debris generated within the femoral canal due to distal stem loosening. These particles likely triggered an inflammatory response, resulting in the development of a periosteal erosive pseudotumor [15].

The recurrence of pseudotumors remains an even more obscure area, with limited understanding of the associated consequences. Cases of recurrence have been reported in the literature [49,50], attributed to various surgical, patient-specific, and implant-related factors. Incomplete intraoperative debridement and residual metal debris have been identified as the primary causes of pseudotumor recurrence [51]. Therefore, achieving complete resection of the mass and any remaining capsule is of utmost importance, with careful attention to safeguarding critical neurovascular structures.

To the best of our knowledge, no cases of pseudotumoral reactions, defined as non-neoplastic inflammatory masses forming in periprosthetic tissues, have been reported in the TAA literature. A modest reaction was documented in a study [17] that reported the presence of a cystic lesion in the fibula four years after TAA. This cyst formation was attributed to mechanical wear of polyethylene caused by misalignment of the components, which increased the peak contact pressures and shear stresses at the bone–implant interface. This can accelerate the wear of the polyethylene spacer and increase the risk of implant failure, subsidence, or loosening over time [52]. Metal hypersensitivity reactions to prosthetic materials in the ankle have been reported in the literature, often presenting with pain and localized symptoms such as swelling or a rash but without the formation of an inflammatory mass [22,53,54]. Anastasio et al. [22] reported a case series with a hypersensitivity reaction to metals post-TAA using a cobalt–chromium implant with a plasma-sprayed coating. The allergic reaction was confirmed by elevated inflammatory markers, positive patch tests, negative rheumatological tests, and clinical improvement following revision with a hypoallergenic implant. However, cases can be asymptomatic despite positive allergy tests: a recent review [20] found that out of 45 patients with metallic devices implanted in their ankles, 19 (42.2%) were allergic to the implant’s metal, but only 14 cases required implant removal. For this reason, the actual risk of implant failure in a patient who tests positive on a cutaneous sensitization test remains low and unpredictable [55,56]. This emphasizes the necessity of including metal allergies in the preoperative assessment process while also drawing attention to the current absence of reliable and validated diagnostic tests for metal hypersensitivity. Existing methods, such as a patch test with a standard set of metals [57] and in vitro assays like the Leukocyte Migration Inhibition Test (LMIT) and the Lymphocyte Transformation Test (LTT) [58], can be valuable when integrated into a comprehensive diagnostic approach. This framework should include a detailed clinical history; symptom evaluation; and exclusion of alternative causes such as infection, rheumatological conditions, and mechanical complications [59]. However, no standardized protocols currently exist for evaluating or managing patients with suspected metal hypersensitivity following foot or ankle surgery, nor are there established guidelines for identifying at-risk patients preoperatively [57]. A reasonable precaution would be to utilize hypoallergenic implants universally to prevent rare but potentially adverse complications.

## 4. Conclusions

In cases of unexplained pain following TAA, although rare, the possibility of a pseudotumor should be considered. This is particularly important today, as the number of TAAs is rapidly increasing and the incidence of complications will inevitably rise. An accurate clinical and radiographic evaluation is crucial as the initial step in identifying this condition, ensuring it is not misdiagnosed. Radiological investigations, including X-ray, CT, and MRI, play a pivotal role in raising suspicion of a pseudotumor, but definitive diagnosis relies on histological examination.

The underlying etiology and pathogenesis of pseudotumors remain unclear and require further studies. Prompt and thorough assessment is essential for guiding appropriate management and preventing potential complications associated with delayed or missed diagnoses.

## Figures and Tables

**Table 1:** Terminology and pathogenesis.

	Etiology	Pathogenetic Mechanism	Clinical Presentation
Metallosis	Accumulation of metal debris	Metal ion release (Co/Cr)	Local tissue necrosis, breakdown of implant materials, and systemic reaction
Debris Disease	Particulate debris from worn implant materials	Inflammatory response with macrophage activation	Osteolysis around implants
ALVAL	Metal hypersensitivity	Immune-mediated reaction characterized by perivascular lymphocyte infiltration	/
Pseudotumor	Metallosis, debris, and metal hypersensitivity	Not clear	A non-neoplastic inflammatory mass within the periprosthetic tissue, periprosthetic bone loss, and loosening of prosthetic components

## Data Availability

Some or all data and models that support the findings of this study are available from the corresponding author upon reasonable request.
